# The Personalised Acne Care Pathway—Recommendations to guide longitudinal management from the Personalising Acne: Consensus of Experts

**DOI:** 10.1016/j.jdin.2021.09.006

**Published:** 2021-10-18

**Authors:** Jerry Tan, Andrew Alexis, Hilary Baldwin, Stefan Beissert, Vincenzo Bettoli, James Del Rosso, Brigitte Dréno, Linda Stein Gold, Julie Harper, Charles Lynde, Diane Thiboutot, Jonathan Weiss, Alison M. Layton

**Affiliations:** a Windsor Clinical Research Inc, Windsor, Ontario, Canada; b Department of Medicine, University of Western Ontario, Windsor Campus, Ontario, Canada; c Weill Cornell Medicine, New York, New York; d Robert Wood Johnson Medical Center, New Brunswick, New Jersey; e The Acne Treatment and Research Center, Brooklyn, New York; f Department of Dermatology, University Hospital Carl Gustav Carus, TU Dresden, Dresden, Germany; g Dermatology Unit – Teaching Hospital, Azienda Ospedaliera, University of Ferrara, Ferrara, Italy; h Thomas Dermatology, Las Vegas, Nevada; i JDR Dermatology Research, Las Vegas, Nevada; j Dermato-cancérology Department, CHU Nantes, University of Nantes, Nantes, France; k Henry Ford Health System, Detroit, Michigan; l Dermatology and Skin Care Center of Birmingham, Birmingham, Alabama; m University of Toronto, Department of Medicine, Markham, Ontario, Canada; n Lynderm Research Inc, Markham, Ontario, Canada; o Department of Dermatology, Pennsylvania State University College of Medicine, Hershey, Philadelphia; p Georgia Dermatology Partners, Snellville, Georgia; q Hull York Medical School, University of York, York, United Kingdom; r Harrogate and District NHS Foundation Trust, Harrogate, United Kingdom

**Keywords:** acne care pathway, acne guidelines, acne patient pathway, acne scarring, acne sequelae, acne vulgaris, consensus, Delphi process, longitudinal management, personalised acne care pathway, personalized care, shared decision-making, truncal acne, HCP, health care professional, PACE, Personalising Acne: Consensus of Experts, PACP, Personalised Acne Care Pathway

## Abstract

**Background:**

Acne is a chronic disease with a varying presentation that requires long-term management. Despite this, the clinical guidelines for acne offer limited guidance to facilitate personalized or longitudinal management of patients.

**Objectives:**

To generate recommendations to support comprehensive, personalized, long-term patient management that address all presentations of acne and its current and potential future burden.

**Methods:**

The Personalising Acne: Consensus of Experts panel consisted of 13 dermatologists who used a modified Delphi approach to reach consensus on statements related to longitudinal acne management. The consensus was defined as ≥75% voting “agree” or “strongly agree.” All voting was electronic and blinded.

**Results:**

Key management domains, consisting of distinct considerations, points to discuss with patients, and “pivot points” were identified and incorporated into the Personalised Acne Care Pathway. Long-term treatment goals and expectations and risk of (or fears about) sequelae are highlighted as particularly important to discuss frequently with patients.

**Limitations:**

Recommendations are based on expert opinion, which could potentially differ from patients' perspectives. Regional variations in health care systems may not have been captured.

**Conclusions:**

The Personalised Acne Care Pathway provides practical recommendations to facilitate the longitudinal management of acne, which can be used by health care professionals to optimize and personalize care throughout the patient journey.

**Capsule Summary**

• Despite the varying presentations and frequently chronic nature of acne, established clinical guidelines offer little guidance to facilitate its personalized and longitudinal patient management
• Based on consensus recommendations, the Personalized Acne Care Pathway panel has developed a Personalized Acne Care Pathway to support comprehensive, personalized, and long-term management of acne

## Introduction

Acne, one of the most common skin conditions treated by dermatologists and other health care professionals (HCPs), generally affects adolescents and young adults, but it can persist later in adulthood despite treatment.^1^, ^2^, ^3^, ^4^, ^5^ Clinically, acne typically presents in various forms, with truncal acne presenting in more than half of the patients with facial involvement.^6^, ^7^, ^8^, ^9^, ^10^, ^11^ Despite its varying presentations and chronic nature, current guidelines offer little guidance to facilitate personalized or longitudinal management of patients.^6^^,^^12^^,^^13^

Personalized care is important in chronic skin conditions, where treatment success is highly dependent on patient adherence to ongoing treatment regimens.^14^ Adherence to treatment can be influenced by numerous factors, including the patients themselves (their characteristics and beliefs), the HCP-patient relationship, and treatment-related factors such as effectiveness, acceptability, side-effect profile, tolerability, frequency of use, duration, and administration routes.^15^ Moreover, disease chronicity and the disease itself (including its anatomic location) play an important role, as can the health care system in which the patient is receiving care (eg, appointment availability and treatment costs).^11^^,^^15^^,^^16^ Low adherence can be due to a perceived lack of response or low treatment satisfaction, highlighting the need for HCPs to consider and discuss patients' concerns, set realistic expectations, and dispel misconceptions throughout their treatment journey.^17^^,^^18^

Ongoing care is also important in managing acne effectively to minimize disease relapse.^19^ It is additionally needed due to the prolonged nature of the treatment, generally taking several months to achieve optimal results.^20^ Patients often misperceive their acne as a short-term condition, emphasizing the need for open and candid communication between HCPs and patients to clarify the long-term nature of management.^21^ Devising and implementing long-term care plans that consider the risk and presence of acne sequelae early in the treatment journey may also mitigate this additional burden on patients.^22^ Management strategies that optimize short-term and long-term outcomes have also been identified by consensus between clinicians and patients as a key research priority, providing a further rationale for developing a care pathway that facilitates longitudinal patient management.^23^

As a part of the 2020-2021 consensus project, the Personalising Acne: Consensus of Experts (PACE) panelists have developed a care pathway based on expert recommendations to support comprehensive, personalized, longitudinal management of acne considering both the current and potential future burden of disease.

## Materials and methods

### Expert panel

The expert panel consisted of 13 dermatologists from Canada (n = 2), France (n = 1), Germany (n = 1), Italy (n = 1) the United Kingdom (n = 1), and the United States (n = 7). Two chairpersons from the main panel oversaw the process and were involved in panel selection and Delphi design.

### The modified Delphi process

**Fig 1:**
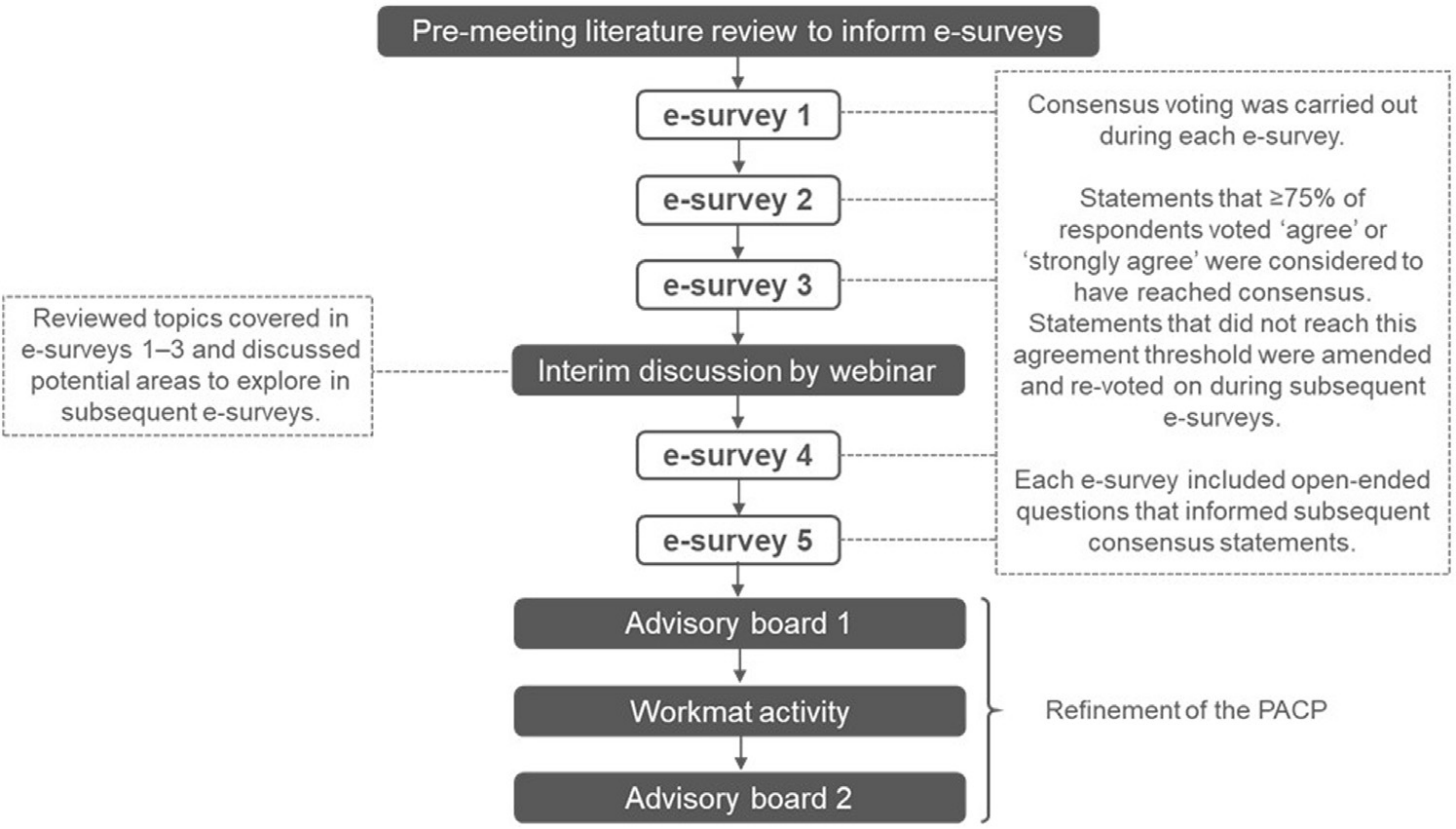
The modified Delphi process used by Personalising Acne: Consensus of Experts panel. *PACP*, Personalised Acne Care Pathway.

The modified Delphi process used by the PACE panel has been described previously.^24^^,^^25^ Between February 2020 and November 2020, 5 e-surveys were conducted to gather information and capture voting responses, with a virtual group meeting held between the third and fourth e-survey. The PACE panelists attended 2 virtual meetings to refine the pathway further and completed a workmat activity in between (Fig 1). An initial literature search was conducted to inform the e-survey content and is described in detail in the Supplementary Material (available via Mendeley at https://data.mendeley.com/datasets/fy6mnvt7t7/1).

### E-survey development and administration

Consensus statements were structured to assess the level of agreement using the response range: “strongly disagree,” “disagree,” “agree,” “strongly agree,” or “unable to answer.” The consensus was defined as ≥75% voting “agree” or “strongly agree.” Some questions were posed as multiple-choice where several responses could be selected, for which results are presented as consensus when chosen by ≥75% of panelists. Some questions were open-ended to allow for the development of consensus statements in a subsequent round of voting. The programming, administration, and response collation of the e-surveys was performed by Ogilvy Health UK to maintain blinding. Longitudinal management and patient types were 2 of the 4 major topics explored and will be the focus of this manuscript. Acne sequelae and truncal acne were also covered and have been previously reported.^24^^,^^25^

## Results

### Definition of consensus recommendations

Consensus statement voting information is provided in parentheses (eg, 12/13 voted “agree” or “strongly agree”). Some panel members occasionally voted “unable to answer”; these votes were not included in the denominator. Complete statements are available in the Supplementary Material. Elements that were considered but not voted on are included in the “Discussion points” below.

### Baseline demographics

When the panelists were asked about clinical practice guidelines, 38% (n = 5) did not find them useful for long-term management strategy, and 62% (n = 8) did not find them useful for the management of different patient types.

### Current challenges and gaps in the longitudinal management of acne

The PACE panel highlighted several challenges in the longitudinal management of acne, including the variable presence of clinical lesions, presence of sequelae, multifactorial pathophysiology, and lack of a definitive treatment target to utilize in a treat-to-target approach. The panelists agreed that there is a need for educational materials for patients on the longitudinal nature of acne management to help them make informed decisions around choice and modification of treatment (13/13).

### Overview of the Personalised Acne Care Pathway

**Fig 2:**
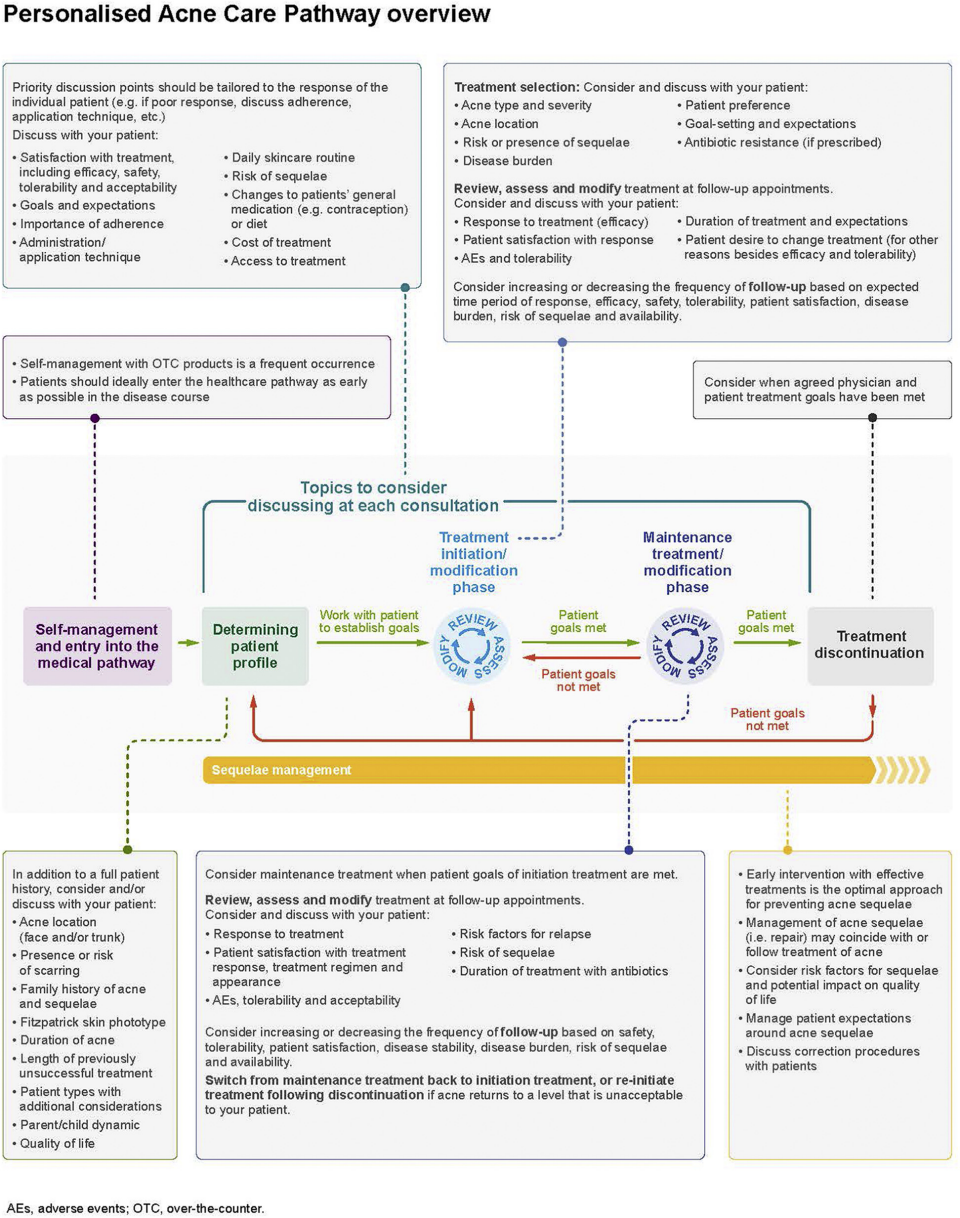
Overview of the PACP. Based on consensus recommendations and discussion from the Personalising Acne: Consensus of Experts panel, 7 key domains consisting of distinct consideration points to discuss with patients and “pivot points” (defined as a central point on which a management decision depends) were identified and incorporated into the PACP. *AE*, Adverse event; *OTC*, over-the-counter; *PACP*, Personalised Acne Care Pathway.

Key management domains were identified across the acne patient journey, consisting of distinct considerations, points to discuss with patients, and “pivot points” (defined as a central point on which a management decision depends). The 7 identified management domains were mapped against the patient journey to form the Personalised Acne Care Pathway (PACP) (Fig 2).

### Self-management and entry into the medical pathway

#### Discussion points

Patients frequently attempt to treat their acne with over-the-counter medications or home remedies recommended by friends and family, or on social media, for some time before presenting to an HCP. Patients eventually present to a dermatology specialist through various pathways, including internet searches, advertising (eg, television or social media), word of mouth, or referral from a primary care practitioner or pharmacist.

### Determining patient profile

Detailed considerations and discussion points for HCPs to determine patient profiles are provided in Table I.

**Table I:** Determining patient profile—detailed considerations and discussion points

Consider
• Acne location ○ Facial vs truncal: assess independently∗ (13/13) • Presence/risk of acne-induced scarring∗ (13/13) • Family history ○ Patients with a family history of acne may be at increased risk of sequelae or uncontrolled disease • Skin phototype ○ For example, acne-induced macular hyperpigmentation in patients with darker skin phototypes (Fitzpatrick scale IV-VI)∗ (13/13) • Duration of acne • Length of previously unsuccessful treatment • Socioeconomic status • Parent/child dynamic

Discuss
• History taking ○ Age and duration of acne ○ Acne location ○ Current acne severity vs “average day” ○ Prior and current treatments and the length of use ○ Prior adverse effects or tolerability issues ○ Level of discomfort/burden/quality of life issues ○ Menstrual cycle irregularities ○ Menstrual flares ○ Signs of hyperandrogenism ○ Family history of acne∗ (13/13) and sequelae ○ Lifestyle triggers ○ Allergies ○ Comorbidities and concomitant medications ○ Use of cosmetics, moisturizers, hair products, and sunscreen∗ (12/13) ○ Knowledge of acne pathogenesis ○ Whey protein use ○ Supplement use ○ Previous endocrinological workup

∗ Indicates topics that were voted on via the Delphi process (Comprehensive list of statements available as Supplementary Material via Mendeley at https://data.mendeley.com/datasets/fy6mnvt7t7/1).

#### Discussion points

Early and comprehensive assessment of the patient's profile is important for optimizing care. Many of the factors concerning patient history could be— and often are—completed before the patient enters the consultation. Several factors such as acne location, patient demographics/specific clinical presentations, medical and family history, skin phototype, and prognostic risk factors can be assessed relatively quickly in a consultation. More time-consuming elements include building and strengthening the HCP-patient rapport, explaining the multifactorial and chronic nature of acne, managing treatment expectations, and ensuring that patients are satisfied with their treatment, feel supported, and understand that the HCP is invested in helping their acne improve.

### Topics to consider for discussion at each consultation

Topics that HCPs should consider discussing with patients at each consultation are provided in Table II.

**Table II:** Topics to consider for discussion at each consultation

Discuss
• Patient-reported satisfaction with acne treatment • Administration/application technique∗ (12/13) • Treatment goals and expectations: ○ It is of paramount importance to discuss long-term treatment expectations with acne patients∗ (13/13) ○ Treatment goals and expectations should be discussed with patients at the first consultation and revisited frequently∗ (13/13) • Efficacy expectations (including timelines)∗ (12/13) • Duration of treatment∗ (12/13) • Adverse effects/tolerability∗ (12/13) • Importance of adherence∗ (12/13) • Daily skincare routine∗ (13/13) • Risk of sequelae • Changes to patient's general medication (eg, contraception) or diet • Cost of treatment • Access to treatment

∗ Indicates topics that were voted on via the Delphi process (Comprehensive list of statements available as Supplementary Material via Mendeley at https://data.mendeley.com/datasets/fy6mnvt7t7/1).

#### Discussion points

The panel identified several patient-related factors, including patient-reported satisfaction, treatment goals and expectations, and risk of (or fears about) sequelae, as important factors to discuss with patients at each consultation.

Goal setting should involve shared decision-making as goals can vary widely among individuals, with the HCP responsible for negotiating with the patient about what is realistically achievable.

HCPs should prioritize discussion topics based on how the individual patient is responding to the prescribed treatment and should enquire about any important changes to their daily routine.

### Treatment initiation/modification phase

The treatment initiation/modification phase is defined as the regimen (which may include a number of different treatments) undertaken to achieve a primary goal before maintenance therapy. Gaps and detailed considerations, discussion points, and pivot points for the treatment initiation/modification phase are included in Table III.

**Table III:** Treatment initiation/modification phase—gaps and detailed considerations, discussion points, and pivot points

Gaps
• There is a need for high-quality evidence for when to escalate, switch, or de-escalate both acne initiation and maintenance treatment∗ (12/13) • Common reasons for nonadherence to initiation and maintenance treatment include treatment intolerance (eg, local irritation)∗ (13/13), perceived lack of efficacy∗ (11/13), and perceived difficulty of use/inconvenience∗ (10/13)

Consider
• Acne type and severity • Acne location (facial and/or truncal acne) • Patient preference • Burden of disease∗ (13/13) • Risk or presence of sequelae • Factors that may affect adherence to treatment (including prior adherence) • Degree of seborrhoea/oiliness • Skin sensitivity • Comorbidities

Discuss
• Goal setting ○ The goals of initiation treatment are to achieve clear or almost-clear skin (depending on patient acceptability) with no new acne lesions and to reduce the risk of sequelae∗ (13/13) ○ Personalized treatment goals depending on the specific impact of acne in certain regions∗ (13/13) ○ Any immediate short-term goals (eg, wedding) • Managing expectations ○ It is of paramount importance to discuss long-term treatment expectations with acne patients∗ (13/13) • Treatment ○ All options should be discussed (including over-the-counter and holistic treatments) based on patient preferences • Antibiotic resistance ○ Prescribers should discuss antibiotic resistance with patients who are prescribed an antibiotic for acne∗ (13/13) • Potential reasons for prior nonadherence to acne medications with patients and adjust their management accordingly∗ (13/13)

Pivot points
• In an ideal situation, patients should be followed up within 3 months where possible during the initiation phase of treatment • Consider increasing the frequency of follow-up when there are safety or tolerability concerns, adherence issues, the patient is particularly anxious or distressed if there is a high risk of sequelae, severe acne, or a lack of efficacy (with current or previous treatments) • Consider decreasing the frequency of follow-up when the treatment is well tolerated, the patient is stable, there is lack of evidence of sequelae development, adherence is good, or for reasons where the patient cannot return for appointments easily (such as going away to college) • Consider the period over which a prescribed treatment is expected to have an effect Review, assess, modify • Switching treatment ○ Consider when there is a lack of response∗ (13/13), the patient is unsatisfied or unhappy with the response∗ (10/13), adverse effects or issues with tolerability occur∗ (12/13), or the patient desires to switch treatment∗ (11/13) • Escalating treatment ○ Consider when there is an inadequate response∗ (13/13) or the patient is unhappy or unsatisfied with the response∗ (12/13) • De-escalating treatment ○ Consider when there are adverse effects/issues with tolerability∗ (10/13) or a patient desire to de-escalate treatment∗ (10/13) • Stopping treatment ○ Consider when treatment goals that were set together with the patient have been met∗ (11/13), satisfactory efficacy outcomes have been achieved∗ (10/13), adverse effects or issues with tolerability occur∗ (10/13), or the patient desires to stop treatment∗ (11/13)

∗ Indicates topics that were voted on via the Delphi process (Comprehensive list of statements available as Supplementary Material via Mendeley at https://data.mendeley.com/datasets/fy6mnvt7t7/1).

#### Discussion points

It was highlighted that during this phase there is also a need to allow for an adjustment period to a medication. As such, the period over which the prescribed medication would be expected to have an effect is an additional factor to consider when determining how frequently to follow-up with patients. In some cases, treatment de-escalation can also be considered in patients when an adequate response has been achieved.

### Maintenance treatment/modification phase

The maintenance phase is defined as the regimen (which may include a number of different treatments) undertaken to maintain the response achieved by initiation treatment (13/13). Gaps and detailed considerations, discussion points, and pivot points for the maintenance treatment/modification phase are included in Table IV.

**Table IV:** Maintenance treatment/modification phase—gaps and detailed considerations, discussion points, and pivot points

Gaps
• There is a need for guidance on the most appropriate time to initiate maintenance therapy∗ (12/13) • Clinical practice guidelines do not provide sufficient guidance on the choice of acne maintenance treatment∗ (12/13) • Clinical practice guidelines do not provide sufficient guidance on when to escalate, switch, or de-escalate acne maintenance treatment∗ (13/13) • There is a need for high-quality evidence for when to escalate, switch, or de-escalate both acne initiation and maintenance treatment∗ (12/13) • Common reasons for nonadherence to initiation and maintenance treatment include treatment intolerance (eg, local irritation)∗ (13/13), perceived lack of efficacy∗ (11/13), and perceived difficulty of use/inconvenience∗ (10/13)

Consider
• Clinical indicators to start maintenance treatment ○ Goals of the initiation phase of treatment achieved ○ Patient satisfaction with treatment response, treatment regimen, and appearance • Duration of treatment with antibiotics • Age/sex of patient (and associated risk of active acne returning) • Patient preference • Completion of the isotretinoin treatment course • Ongoing cost of treatment

Discuss
• Goal setting ○ The goals of the maintenance phase are to maintain clear/almost-clear skin achieved during the initiation phase, to prevent acne from returning to a level that is unacceptable to the patient, and to reduce the risk of acne sequelae∗ (13/13)

Pivot points
• In an ideal situation, patients should be followed up at least twice a year during the maintenance phase of treatment • Consider increasing the frequency of follow-up when there are safety or tolerability concerns or adherence issues, when the patient is particularly anxious or needs encouragement, if sequelae develop, or when there is a lack of efficacy or return of active acne to a level unacceptable to the patient • Consider decreasing the frequency of follow-up when the patient is stable, when the agreed treatment goals have been met, or for reasons where the patient cannot return easily for appointments (such as going away to college) Review, assess, modify • Switching treatment ○ Consider when there is a lack of response∗ (10/13), the patient is unsatisfied or unhappy with the response∗ (10/13), there are adverse effects/issues with tolerability∗ (12/13), or there is poor acceptability of treatment (eg, oiliness, odor, and bleaching/staining)∗ (10/13) • Switching from maintenance back to initiation treatment ○ Consider when there is a lack of response (eg, acne returning to premaintenance state)∗ (13/13) or the patient is unsatisfied or unhappy with the response∗ (11/13)

∗ Indicates topics that were voted on via the Delphi process (Comprehensive list of statements available as Supplementary Material via Mendeley at https://data.mendeley.com/datasets/fy6mnvt7t7/1).

#### Discussion points

Tele-visits are an acceptable platform for follow-up visits if patients are progressing well on maintenance treatment.

### Treatment discontinuation

Treatment discontinuation can be considered when the treatment goals that have been mutually developed and agreed by the clinician and patient have been met.

### Sequelae management

Detailed considerations, discussion points, and pivot points for sequelae management are included in Table V.

**Table V:** Sequelae management—detailed considerations, discussion points, and pivot points

Consider
• Risk factors for sequelae • Impact on quality of life • Skin phototype

Discuss
• Acne sequelae should be discussed with patients at the first consultation and revisited frequently • Managing expectations: ○ Discuss their concerns around the effect of their disease∗ (11/13) ○ Discuss their concerns around treatment∗ (10/13) ○ Discuss their expectations from treatment∗ (11/13) ○ Highlight that improvement may only be observed in the long term∗ (10/13) ○ Be realistic with them about outcomes∗ (11/13) ○ Emphasize the need for control of active acne to reduce the risk of developing sequelae∗ (13/13) ○ Emphasize the role of modifiable risk factors (eg, lesion excoriation, adherence to medication) in reducing the risk of developing sequelae∗ (13/13) ○ Discuss management options for sequelae∗ (10/13) • Discuss correction procedures with patients

Pivot points
• Consider whether the patient needs time for existing lesions to heal

∗ Indicates topics that were voted on via the Delphi process (Comprehensive list of statements available as Supplementary Material via Mendeley at https://data.mendeley.com/datasets/fy6mnvt7t7/1).

#### Discussion points

Sequelae management has been previously reported in detail.^25^

### Additional considerations for patients with specific clinical presentations

Patient types that require additional considerations for their acne management are provided in Table VI.

**Table VI:** Additional factors to consider for patients with specific clinical presentations

Patients with specific clinical presentations	Consider
Children aged <10 years∗ (12/13)	• Lack of established skin care routine and potential hormonal conditions∗ (11/13)
Patients with darker skin phototypes (Fitzpatrick scale IV-VI)∗ (13/13)	• Acne-induced macular hyperpigmentation∗ (13/13)
	• Additional hyperpigmentation caused by irritation from topical medication∗ (11/13)
	• Potential inappropriate use of bleaching creams∗ (10/13)
	• Cultural cosmetic practices that may influence acne (eg, the use of oils in hair)∗ (11/13)
Patients with hormonal conditions∗ (13/13)	• Difficulty in counteracting the effects of exogenous androgens∗ (12/13)
	• The need for interdisciplinary management with an endocrinologist∗ (11/13)
	• Requirements for laboratory examinations∗ (12/13)
Heavy exercisers/athletes∗ (11/13)	• Potential use of anabolic steroids or supplements∗ (12/13)
Patients at risk of psychiatric issues∗ (10/13)	• The potential to be engaging in harsh cleaning routines or excoriation/manipulation of lesions∗ (12/13)
	• Drug-induced acne∗ (10/13)
	• The need for interdisciplinary management with a psychiatrist or other allied health care professionals∗ (12/13)
Women who are pregnant or breastfeeding (11/13)	*Recommendations for considerations not explored further*
Transgender patients∗ (12/13)	*Recommendations for considerations not explored further*
Patients with medication-induced acne∗ (10/13)	• Patients with medication-induced acne (including those receiving cancer treatment, eg, epidermal growth factor receptor inhibitors)∗ (10/13)
Adult male patients	• Steroid or supplement-induced acne∗ (11/13)
Adult female patients	• Use of hormonal treatments (eg, contraception)∗ (13/13)
	• Potential polycystic ovary syndrome∗ (12/13)
	• Use of make-up and other cosmetic skincare products∗ (11/13)
	• Pregnancy and lactation∗ (12/13)
Specific populations of acne patients may benefit from an interdisciplinary approach to management (11/12)	

∗ Indicates topics that were voted on via the Delphi process (Comprehensive list of statements available as Supplementary Material via Mendeley at https://data.mendeley.com/datasets/fy6mnvt7t7/1).

#### Discussion points

All patients should be managed using an individualized approach; however, the panel identified a number of specific clinical presentations that may require additional considerations.

## Discussion

This PACP represents a consolidated effort to provide expert recommendations for the longitudinal management of acne. It incorporates patient-centered goals, reviewing, assessing, and modifying treatment, a transition from initiation to maintenance therapy, and guidance on how to manage patients in cases of relapse or remission. Alternative terminology has been previously used to describe the treatment initiation/modification phase in the PACP (eg, induction treatment); however, the recommendations for this treatment phase remain the same.^6^

Current clinical guidelines for acne recommend treatment primarily based on disease-related factors, such as the type of acne and severity of disease, at fixed time points during the treatment journey, and almost exclusively focus on facial acne, with little guidance regarding the treatment of truncal acne.^6^^,^^12^^,^^13^ The PACP complements clinical guidelines by addressing all presentations of acne and providing a framework to manage patients in a dynamic and holistic way, taking into consideration treatment- and patient-related factors as well as the current and future burden of disease. The PACE panel has previously provided recommendations to improve the management of truncal acne and acne sequelae.^24^^,^^25^ These recommendations highlighted the importance of addressing truncal acne and acne sequelae as early as possible in the patient journey to mitigate the additional physical and psychosocial burden imposed on patients.^26^^,^^27^ In addition, given that understanding patient expectations and mutually determined goals is inextricably linked to a positive HCP-patient relationship, the PACP is designed to cultivate a shared commitment to care personalized to each patient.^15^^,^^17^^,^^28^ As such, it is important to emphasize that the PACP is not intended to act as a substitute for clinical guidelines and advise on specific treatment recommendations but instead is intended to provide guidance in optimizing the process of care in clinical practice.

Patient satisfaction with treatment is one of the most important aspects to consider in ongoing management to improve adherence to treatment regimens; however, this is multifactorial and includes whether patients are satisfied with the improvement in their acne with the treatment regimen and whether they are satisfied with their appearance.^15^^,^^17^^,^^18^ Currently, the majority of treatment algorithms in national and regional clinical management guidelines do not incorporate patient-oriented treatment goals or patient satisfaction as an outcome.^6^^,^^12^^,^^13^^,^^29^, ^30^, ^31^, ^32^, ^33^, ^34^, ^35^, ^36^, ^37^, ^38^, ^39^ Thus the PACE panel has recommended addressing patient satisfaction in consultations to improve adherence and ultimately improve patient outcomes.

A limitation is that the recommendations outlined in the PACP are based on the experiences of the expert panel and reflect HCP perspectives on the important topics to discuss with patients, which could potentially differ from patients' perspectives.^28^ In addition, although the PACP integrates recommendations from an international group of experts, it only represents the health care systems in which the PACE panel has the experience and may not account for nuances in other regions.^40^^,^^41^ Similar strengths and limitations of the Delphi method apply, as previously reported.^24^^,^^25^

The PACP represents a simple, clear, and comprehensive overview of the acne patient journey, which emphasizes the multistep nature of acne management. Future applications for the PACP could include the development of iterations of the pathway for different audiences, for example, a version for patients, primary care practitioners, or nurses. This could be particularly helpful for patients (especially adolescents) to emphasize that their journey with acne could be an ongoing and dynamic process. Patient cases to demonstrate the use of the pathway could also be a valuable tool and have been used previously in other dermatologic conditions, such as rosacea.^42^ Practical applications of the PACP include potential use by HCPs as an “acne care road map” while treating patients to accompany the patient through their acne journey and to determine individual pivot points in partnership with the patient. Feedback on the use of the PACP in clinical practice will be required to inform future updates and the development of practical tools to facilitate its use. Digital forms of the road map could also be developed to allow real-time support and feedback for HCPs during discussions with patients.

## Conclusion

The PACE panel has developed the PACP to provide practical and actionable recommendations to facilitate personalized, longitudinal management of patients with acne. These recommendations can inform local guideline development and patient consultations, thus helping to optimize and personalize care throughout the patient journey.

## Conflicts of interest

All panel members received honoraria from Galderma for participating in this project. Dr Tan has acted as an advisor, consultant, investigator, and/or speaker and has received grants/honoraria from Bausch, 10.13039/501100009754Galderma, 10.13039/100004319Pfizer, Almirall, Boots/10.13039/100005153Walgreens, Botanix, Cipher Pharmaceuticals, Novan, 10.13039/100004336Novartis, Promius, 10.13039/501100013671Sun Pharma, Vichy, CeraVe, Cutera, and La Roche-Posay. Dr Alexis has received grant/research support from LEO Pharma, 10.13039/100004336Novartis, Almirall, 10.13039/100002491Bristol-Myers Squibb, 10.13039/100002429Amgen, Menlo, 10.13039/501100009754Galderma, 10.13039/100011284Valeant (10.13039/501100014565Bausch Health), Cara, and Arcutis; has acted as a consultant for LEO Pharma, Novartis, Menlo, Galderma, Pfizer, Sanofi-Regeneron, Dermavant, Unilever, Beiersdorf, Valeant, L'Oreal, Bristol-Myers-Squibb, Menlo, Scientis, Bausch Health, UCB, Foamix, Cassiopea, Arcutis, Janssen, Allergan, Almirall, AbbVie, and Sol-Gel; and has acted as a speaker (unbranded) for Regeneron, SANOFI-Genzyme, Pfizer, and AstraZeneca. Dr Baldwin has acted as an investigator, consultant, and/or speaker for Almirall, Bausch Health, Cassiopea, EPI Health, Galderma, La Roche-Posay, L'Oréal, Mayne Pharma, Sol-Gel, Sun Pharma, and Vyne. Dr Beissert has acted as an advisory board member for AbbVie Deutschland GmbH & Co. KG, Actelion Pharmaceuticals Deutschland GmbH, Amgen GmbH, Celgene GmbH, Galderma Laboratorium GmbH, Janssen-Cilag GmbH, LEO Pharma GmbH, Lilly Deutschland GmbH, Novartis Pharma GmbH, MSD Sharp & Dohme GmbH, Menlo Therapeutics, Sanofi-Aventis Deutschland GmbH, Pfizer Pharma GmbH, UCB Pharma GmbH, and has received speaker honorarium from Novartis Pharma GmbH, AbbVie Deutschland GmbH & Co. KG, MSD Sharp & Dohme GmbH, Pfizer Pharma GmbH, Janssen-Cilag GmbH, Galderma Laboratorium GmbH, Celgene GmbH, La Roche-Posay Laboratoire Pharmaceutique, Actelion Pharmaceuticals Deutschland GmbH, GlaxoSmithKline GmbH & Co. KG, Bristol-Myers Squibb GmbH & Co. KGaA, Sanofi-Aventis Deutschland GmbH, Almirall-Hermal GmbH, and Sandoz/HEXAL AG. Dr Bettoli has acted as consultant, advisory board member, and research investigator and has received honoraria from AbbVie, Baiersdorf, Bioderma, Biogena, Difa-Cooper, Galderma, GSK, ICF, LEO Pharma, L'Oréal, Meda, Menarini – Relife, Mylan, Novartis, Pharcos-Biodue, and UCB Pharma and has received research support (funds to institution) from 10.13039/100006483AbbVie. Dr Del Rosso has acted as a research investigator, consultant, and/or speaker for Almirall, Bausch Health (Ortho Dermatology), BiopharmX, EPI Health, Galderma, LEO Pharma, Mayne Pharma, Sol-Gel, Sonoma, Sun Pharma, and Vyne Therapeutics (Foamix). Dr Dréno has acted as a consultant for Galderma. Dr Stein Gold has acted as an investigator/advisor and/or speaker for Galderma, Ortho Derm, Sun Pharma, Sol-Gel, Foamix, Novartis, and Almirall. Dr Harper has acted as a consultant for Almirall, BioPharmX, Cassiopea, Cutera, EPI, Galderma, Ortho, Sol-Gel, Sun Pharma, and Vyne Therapeutics. Dr Lynde has acted as a PI, speaker, and consultant for Cipher Pharma, Bausch Health, Galderma, Johnson & Johnson, GSK, and Valeant. Dr Thiboutot has acted as a consultant for Cassiopea, Galderma, and Novartis. Dr Weiss has acted as an investigator/advisor and/or speaker for Galderma, Ortho Derm, Foamix, Novartis, Almirall, Dr. Reddy's, and EPI Health. Dr Layton has acted as an advisor or consultant, been the chief investigator for research (funded to institution), and/or received honoraria for unrestricted educational events from Almirall, Galderma, La Roche-Posay, LEO Pharma, L'Oréal, Cipher, Mylan, Novartis, Proctor and Gamble, GSK, and Origimm.

## References

[1] Bhate K., Williams H.C. (2013). Epidemiology of acne vulgaris. Br J Dermatol.

[2] Layton A.M., Thiboutot D., Tan J. (2021). Reviewing the global burden of acne: how could we improve care to reduce the burden?. Br J Dermatol.

[3] Ridd M.J. (2017). The management of acne in primary care. Br J Dermatol.

[4] Schäfer T., Nienhaus A., Vieluf D., Berger J., Ring J. (2001). Epidemiology of acne in the general population: the risk of smoking. Br J Dermatol.

[5] Collier C.N., Harper J.C., Cafardi J.A. (2008). The prevalence of acne in adults 20 years and older. J Am Acad Dermatol.

[6] Nast A., Dréno B., Bettoli V. (2016). European evidence-based (S3) guideline for the treatment of acne - update 2016 - short version. J Eur Acad Dermatol Venereol.

[7] Del Rosso J.Q., Bikowski J.B., Baum E. (2007). A closer look at truncal acne vulgaris: prevalence, severity, and clinical significance. J Drugs Dermatol.

[8] Del Rosso J.Q., Stein-Gold L., Lynde C., Tanghetti E., Alexis A.F. (2019). Truncal acne: a neglected entity. J Drugs Dermatol.

[9] Tan J.K., Tang J., Fung K. (2008). Prevalence and severity of facial and truncal acne in a referral cohort. J Drugs Dermatol.

[10] Dréno B., Jean-Decoster C., Georgescu V. (2016). Profile of patients with mild-to-moderate acne in Europe: a survey. Eur J Dermatol.

[11] Poli F., Auffret N., Leccia M.T., Claudel J.P., Dréno B. (2020). Truncal acne, what do we know?. J Eur Acad Dermatol Venereol.

[12] Zaenglein A.L., Pathy A.L., Schlosser B.J. (2016). Guidelines of care for the management of acne vulgaris. J Am Acad Dermatol.

[13] Asai Y., Baibergenova A., Dutil M. (2016). Management of acne: Canadian clinical practice guideline. CMAJ.

[14] Abbott P. (2017). Patient-centred health care for people with chronic skin conditions. Br J Dermatol.

[15] Eicher L., Knop M., Aszodi N., Senner S., French L.E., Wollenberg A. (2019). A systematic review of factors influencing treatment adherence in chronic inflammatory skin disease - strategies for optimizing treatment outcome. J Eur Acad Dermatol Venereol.

[16] Kvarnström K., Airaksinen M., Liira H. (2018). Barriers and facilitators to medication adherence: a qualitative study with general practitioners. BMJ Open.

[17] Alsubeeh N.A., Alsharafi A.A., Ahamed S.S., Alajlan A. (2019). Treatment adherence among patients with five dermatological diseases and four treatment types - a cross-sectional study. Patient Prefer Adherence.

[18] Hayran Y., İncel Uysal P., Öktem A., Aksoy G.G., Akdoğan N., Yalçın B. (2021). Factors affecting adherence and patient satisfaction with treatment: a cross-sectional study of 500 patients with acne vulgaris. J Dermatolog Treat.

[19] Dreno B., Bordet C., Seite S., Taieb C. (2019). Acne relapses: impact on quality of life and productivity. J Eur Acad Dermatol Venereol.

[20] Ozolins M., Eady E.A., Avery A. (2005). Randomised controlled multiple treatment comparison to provide a cost-effectiveness rationale for the selection of antimicrobial therapy in acne. Health Technol Assess.

[21] Ip A., Muller I., Geraghty A.W.A., Platt D., Little P., Santer M. (2021). Views and experiences of people with acne vulgaris and healthcare professionals about treatments: systematic review and thematic synthesis of qualitative research. BMJ Open.

[22] Tan J., Tanghetti E., Baldwin H., Stein Gold L., Lain E. (2019). The role of topical retinoids in prevention and treatment of atrophic acne scarring: understanding the importance of early effective treatment. J Drugs Dermatol.

[23] Layton A., Eady E.A., Peat M. (2015). Identifying acne treatment uncertainties via a James Lind Alliance Priority Setting Partnership. BMJ Open.

[24] Tan J, Alexis A, Baldwin H (2021). Gaps and recommendations for clinical management of truncal acne from the personalising acne: Consensus of Experts panel. JAAD Int.

[25] Layton A, Andrew A, Baldwin B (2021). Identifying gaps and providing recommendations to address shortcomings in the investigation of acne sequelae by the personalising acne: Consensus of Experts panel. JAAD Int.

[26] Hassan J., Grogan S., Clark-Carter D., Richards H., Yates V.M. (2009). The individual health burden of acne: appearance-related distress in male and female adolescents and adults with back, chest and facial acne. J Health Psychol.

[27] Hayashi N., Miyachi Y., Kawashima M. (2015). Prevalence of scars and “mini-scars”, and their impact on quality of life in Japanese patients with acne. J Dermatol.

[28] Mühlbacher A.C., Juhnke C. (2013). Patient preferences versus physicians' judgement: does it make a difference in healthcare decision making?. Appl Health Econ Health Policy.

[29] Eichenfield L.F., Krakowski A.C., Piggott C. (2013). Evidence-based recommendations for the diagnosis and treatment of pediatric acne. Pediatrics.

[30] Bagatin E., Florez-White M., Arias-Gomez M.I., Kaminsky A. (2017). Algorithm for acne treatment: Ibero-Latin American consensus. An Bras Dermatol.

[31] Le Cleach L., Lebrun-Vignes B., Bachelot A. (2017). Guidelines for the management of acne: recommendations from a French multidisciplinary group. Br J Dermatol.

[32] Szepietowski J., Kapińska-Mrowiecka M., Kaszub A. (2012). Acne vulgaris: pathogenesis and treatment. Consensus of the Polish Dermatological Society. Przegl Dermatol.

[33] López-Estebaranz J.L., Herranz-Pinto P., Dréno B. (2017). Consensus-based acne classification system and treatment algorithm for Spain. Actas Dermosifiliogr.

[34] Acne vulgaris: management (draft for consultation, December 2020). National Institute for Health and Care Excellence. https://www.nice.org.uk/guidance/GID-NG10109/documents/draft-guideline.

[35] Oon H.H., Wong S.N., Aw D.C.W., Cheong W.K., Goh C.L., Tan H.H. (2019). Acne management guidelines by the Dermatological Society of Singapore. J Clin Aesthet Dermatol.

[36] Terapia topica dell'acne lieve e moderat, 2015. Societa Italiana Di Dermatologia Medica, Chirurgica, Estetica E Delle Malattie Sessualmente Trasmesse (SIDeMaST). https://www.pacinimedicina.it/wp-content/uploads/acne-lieve-sidemast-38710.pdf.

[37] Hayashi N., Akamatsu H., Iwatsuki K. (2018). Japanese Dermatological Association Guidelines: guidelines for the treatment of acne vulgaris 2017. J Dermatol.

[38] Goh C.L., Abad-Casintahan F., Aw D.C. (2015). South-East Asia study alliance guidelines on the management of acne vulgaris in South-East Asian patients. J Dermatol.

[39] Nast A., Bayerl C., Borelli C. (2010). S2k-guideline for therapy of acne. Article in German. J Dtsch Dermatol Ges.

[40] Fink-Hafner D., Dagen T., Doušak M., Novak M., Hafner-Fink M. (2019). Delphi methods: strengths and weaknesses. Metodološki Zv.

[41] Tan J., Wolfe B., Weiss J. (2012). Acne severity grading: determining essential clinical components and features using a Delphi consensus. J Am Acad Dermatol.

[42] Schaller M., Almeida L.M.C., Bewley A. (2020). Recommendations for rosacea diagnosis, classification and management: update from the global ROSacea COnsensus 2019 panel. Br J Dermatol.

